# The interplay of prefrontal and sensorimotor cortices during inhibitory control of learned motor behavior

**DOI:** 10.3389/fneng.2012.00017

**Published:** 2012-07-25

**Authors:** Selina C. Wriessnegger, Günther Bauernfeind, Kerstin Schweitzer, Silvia Kober, Christa Neuper, Gernot R. Müller-Putz

**Affiliations:** ^1^Laboratory of Brain-Computer Interfaces, Institute for Knowledge Discovery, Graz University of Technology, A-8010 GrazSteiermark, Austria; ^2^Department of Psychology, University of Graz, A-8010 GrazSteiermark, Austria

**Keywords:** motor learning, response inhibition, anterior prefrontal cortex (APFC), PFC, motor cortex, fNIRS

## Abstract

In the present study inhibitory cortical mechanisms have been investigated during execution and inhibition of learned motor programs by means of multi-channel functional near infrared spectroscopy (fNIRS). fNIRS is an emerging non-invasive optical technique for the *in vivo* assessment of cerebral oxygenation, concretely changes of oxygenated [oxy-Hb], and deoxygenated [deoxy-Hb] hemoglobin. Eleven healthy subjects executed or inhibited previous learned finger and foot movements indicated by a visual cue. The execution of finger/foot movements caused a typical activation pattern namely an increase of [oxy-Hb] and a decrease of [deoxy-Hb] whereas the inhibition of finger/foot movements caused a decrease of [oxy-Hb] and an increase of [deoxy-Hb] in the hand or foot representation area (left or medial somatosensory and primary motor cortex). Additionally an increase of [oxy-Hb] and a decrease of [deoxy-Hb] in the medial area of the anterior prefrontal cortex (APFC) during the inhibition of finger/foot movements were found. The results showed, that inhibition/execution of learned motor programs depends on an interplay of focal increases and decreases of neural activity in prefrontal and sensorimotor areas regardless of the effector. As far as we know, this is the first study investigating inhibitory processes of finger/foot movements by means of multi-channel fNIRS.

## Introduction

In daily life successful human behavior strongly depends on learning and inhibition of inappropriate behavior. Specifically inhibitory control is an essential function to provide appropriate preparation and online control of required motor programs. Furthermore a fine balance between activation and inhibition is necessary for preparation of movement, initiation, motor control, and timely inhibition of the act. There are a lot of studies focusing on the question about the neural correlate of effective inhibition or suppression of behavior. Most of them used experimental paradigms like GO/NOGO (Rubia et al., 2003; Herrmann et al., 2005; Simmonds et al., 2008) tasks or STOP-Signal (Boecker et al., 2007; Tabu et al., 2011, 2012) paradigms to investigate inhibition processes. The differences between the tasks are that the GO/NOGO paradigm requires a response selection process, namely execute or inhibit a motor response, triggered by a go or a no-go-stimulus. On the other hand, in the stop task the stop signal requires withholding or stopping an already triggered motor response. For example, the results of a meta-analysis using 11 studies of event-related functional magnetic resonance imaging (fMRI) during GO/NOGO task have shown that the pre-supplementary motor area (pre-SMA) and the prefrontal-parietal circuits are crucial for response inhibition (Simmonds et al., 2008). More evidence for the involvement of the prefrontal cortex (PFC) in response inhibition came from Rubia et al. (2000, 2001, 2003) who found predominantly right hemispheric PFC activations. For example in a stop-signal study (Rubia et al., 2003) they found different activation patterns for successful and failed stopping. The right inferior PFC was correlated with successful inhibition and bilateral inferior parietal cortices were associated with failed inhibition. Whereas most fMRI studies investigated only manual response inhibition the recent study of Tabu et al. (2012) investigated also the brain representation of foot stop-signal task for the first time. They found common activation patterns of prefrontal areas (pre-SMA and bilateral ventrolateral PFC) for hand and foot stop signal tasks.

Beside a lot of fMRI studies there are also some fNIRS studies focusing on the role of PFC activation during cortical inhibition (Boecker et al., 2007; Kono et al., 2007). Functional near infrared spectroscopy (fNIRS) is a non-invasive optical imaging technique to quantify cortical activity. fNIRS allows measuring the oxygenation (haemoglobin concentration) in the cerebral cortex, which is strongly correlated to the fMRI Blood-Oxygen-Level-Dependent (BOLD) signal (Strangman et al., 2002; Steinbrink et al., 2006). Even though fNIRS has lower spatial resolution than fMRI, it has the advantage of providing information about two parameters, namely oxygenated- (oxy-Hb) and deoxygenated (deoxy-Hb) hemoglobin. As fNIRS measures changes in oxy-Hb and deoxy-Hb and consequential total hemoglobin (tot-Hb) concentration, this approach allows also to draw conclusions about changes in neurovascular parameters like cerebral metabolic rate of oxygen (CMRO2), cerebral blood flow (CBF), and cerebral blood volume (CBV) (Malonek and Grinvald, 1996; Malonek et al., 1997; Wolf et al., 2002). Furthermore it is not sensitive to motion artifacts, is portable and can be easily used with children and patients (Strangman et al., 2002; Wolf et al., 2002).

For example Herrmann et al. (2005) replicated previous findings from fMRI-studies using fNIRS in a GO/NOGO paradigm. He found significantly higher increases of oxy-Hb and decreases of deoxy-Hb concentration during inhibition phases in the inferior part of the PFC.

It is known that successful behavior also requires appropriate retrieval of acquired motor programs or inhibition of learned actions. That is, activation and deactivation or inhibition of brain regions representing these actions. Another paradigm investigating cortical inhibition used by Hummel et al. (2002, 2004) is similar to common GO/NOGO tasks but has no time pressure and is additionally based on previous learning processes. Hummel et al. (2002) showed that acquired motor behavior is a context-dependent interaction of execution and inhibition of learned motor programs. Inhibition was associated with a decrease in motor cortical excitability below the resting state and was additionally correlated with a task-related increase of 11–13 Hz oscillatory activity on the electroencephalogram (EEG). In a later study they used fMRI to investigate inhibition of learned motor programs (Hummel et al., 2004). They found that the inhibitory changes were characterized by negative BOLD responses in an extended cerebro-cerebellar network of sensorimotor structures with a predominant role of the PFC. Such PFC activation was also found in the fNIRS study by Boecker et al. (2007) reporting a substantial activation increase in the right PFC during inhibition of already initiated responses.

In the present study we applied fNIRS to healthy subjects performing a paradigm comparable to that used by Hummel et al. (2004). We investigated bidirectional inhibition-activation processes during execution/inhibition of learned motor programs executed by hand and foot. The aim of the present study was two-fold: first we wanted to replicate the findings of Hummel et al. (2004) using fMRI with multichannel fNIRS. Secondly, we investigated hemodynamic changes of response inhibition during foot movements. To our knowledge the inhibition of learned motor programs executed by hand and foot has never been investigated with multichannel fNIRS.

## Materials and methods

### Participants

Investigations were carried out on a group of 11 voluntary healthy subjects (four males, seven females) aged from 22 to 37 years (27.3 ± 3.9, mean ± SD). All subjects were right-handed and had normal or corrected to normal vision. Hand performance was assessed with the “Hand Dominance Test” (HDT) by Steingrüber and Lienert (1971). This test comprises three dexterity tasks, each to be performed with maximal speed and precision over 15 s, separately for the right and left-hand (tracing lines, dotting circles, and dotting squares). In this regard, dominance refers to the performance advantage of one hand relative to the other. All experiments were in compliance with the World Medical Association Declaration of Helsinki. The protocol was approved by the Ethics committee of the Medical University of Graz and the subjects gave informed written consent before the experiment.

### Experimental paradigm

Three weeks prior to the experiment, subjects were instructed to train themselves on six sequences of right hand finger and right foot movements of two different task complexities at home. In Table 1 the three experimental blocks are described in detail, divided by type of limb (finger or foot), presentation modality (activation, inhibition), sequence type (easy/difficult), number of trials and total duration of each block (Table 1). The necessary resources, a keyboard for finger movements and a template to train foot movements, were provided to the participants. Prior the experimental session the success of the training was tested. Only subjects, who successfully completed the test, meaning that they executed the requested finger/foot movements without errors, performed the experiment.

**Table 1:** **Experimental blocks**.

**Block**	**Limb**	**Presentation modality**	**#Trials**	**Duration**
1	finger	execution: 12 easy^*a*^, 12 difficult^*b*^ inhibition: 12 easy, 12 difficult 2 new	50	13 min
2	foot	execution: 12 easy, 12 difficult inhibition: 12 easy, 12 difficult 2 new	50	13 min
3	finger	execution: 6 easy, 6 difficult inhibition: 6 easy, 6 difficult 2 new	52	13 min
	foot	execution: 6 easy, 6 difficult inhibition: 6 easy, 6 difficult 2 new		

a easy sequences: 1-2-3-4-1-2-3-4 1-1-2-2-3-3-4-4 1-3-2-4-1-3-2-4

b difficult sequences: 4-1-3-2-1-2-3-1 3-2-1-4-3-4-1-3 4-2-1-4-3-1-4-2

During the experimental sessions all subjects were seated in a comfortable arm-chair in front of a TFT monitor. The distance between the participants and the screen was about 120 cm. To avoid artifacts, the participants were instructed to relax as much as possible during the measurement. The study consisted of three sessions (Table 1): a finger movement session (indicated by a picture of a hand), a foot movement session (indicated by a picture of a foot), and a session with randomized finger and foot movements. Sessions were presented blockwise in the described order (Table 1). Within each block 50% of trials required inhibition and 50% execution. The sequences in the blocks were randomly presented.

**Figure 1:**
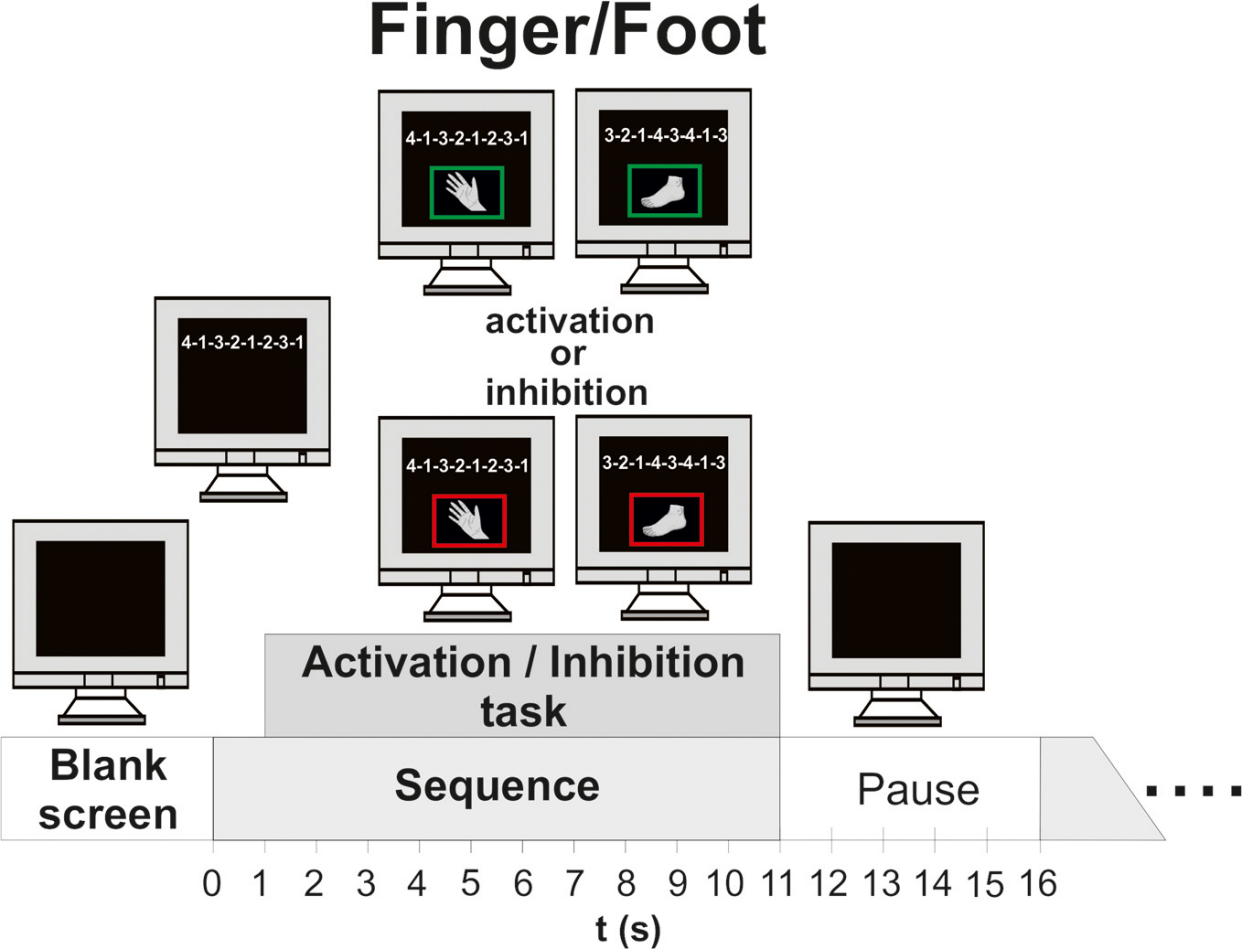
**Time course of one experimental trial (finger/foot, execution/inhibition). Left side:** timing of finger movement execution/inhibition; **Right side**: timing of foot movement execution/inhibition.

**Figure 2:**
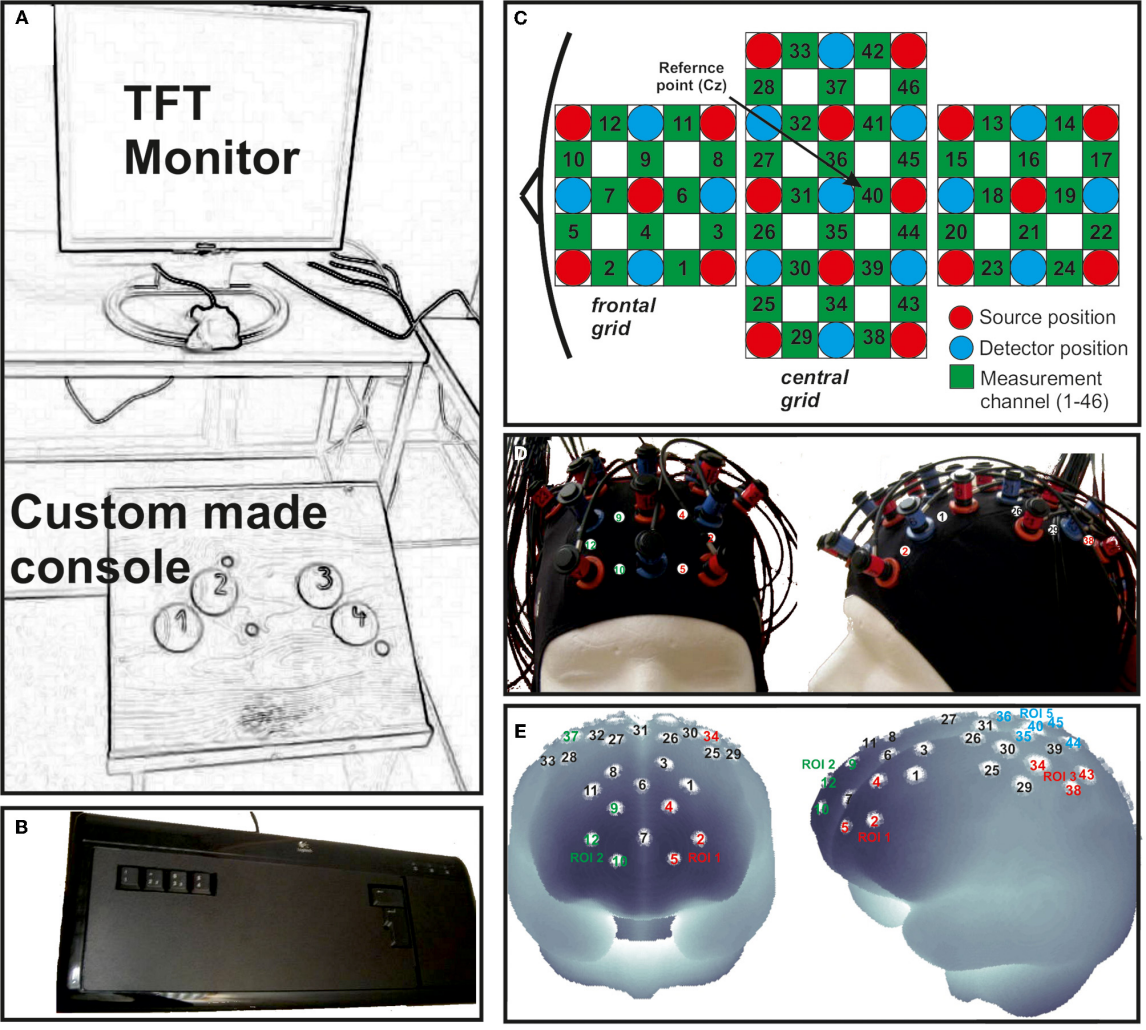
**(A)** Custom made console used for foot movement responses positioned in front of a TFT monitor. **(B)** Modified keyboard for finger movement responses. **(C)** Schematic illustration of the multi-channel arrays (46 channels, two 3 × 3 grids and one 3 × 5 grid) covering frontal, central and parietal regions. **(D)** fNIRS cap with mounted optodes. **(E)** Projections of the fNIRS channel positions on the cortical surface. Positions are overlaid on a MNI-152 compatible canonical brain which is optimized for fNIRS analysis.

During the finger movement session subjects had to execute or inhibit 48 sequences of right hand finger movements presented on the monitor. In order to indicate if execution or an inhibition task was required a green (execution) or a red (inhibition) frame was shown around the picture of the hand one second after sequence presentation. One sequence consisted of eight movements [presented on the screen as a sequence of eight numbers (digits 1–4)] and lasted 10 s. The fingers were labeled corresponding to these digits as follows: the index finger “1,” the middle finger “2,” the ring finger “3,” the little finger “4.” During the execution task the subjects were instructed to perform the requested sequence (e.g., 4-1-3-2-1-2-3-1) on a modified keyboard until the screen turns black. The average frequency of finger (Figure 2B) tapping was about 25.03, resulting in about three sequences. During the inhibition task they should avoid any button press. After one trial a pause of 5 s followed. Additionally to the well trained sequences, two new sequences (1 execution, 1 inhibition) were presented in order to maintain the subject's attention. So the finger movement session consisted of 50 trials. A detailed description of the timing of one trial is given in Figure 1.

During the foot movement session subjects had to execute or inhibit foot movement sequences on a custom made console (Figure 2A). The average frequency of foot tapping was 21.18, resulting in nearly three full sequences. Apart from that, the timing and number of trials were the same as in the finger movement block. Again, in order to indicate if execution or inhibition was required a green (execution) or a red (inhibition) frame was shown around the picture of the foot.

Finally, in the session with randomized finger and foot movements the subjects had to execute or inhibit 24 fingers and foot movement sequences in random order. In contrast to the finger and foot block, four new sequences occurred, so the block consisted of 52 trials. Between the blocks the subjects had short breaks of about 5 min (see Figure 1).

### Data acquisition and processing

To record brain oxygenation a multichannel commercial fNIRS system (ETG-4000, Hitachi Medical Co., Japan), which is based on the continuous wave principle was used. The sampling rate was set to 10 Hz. The multi-channel system measures the change of [oxy-Hb] and [deoxy-Hb]^1^ in the unit of m(mol/l) × mm (further denoted as mM mm) and consisted of 15 photo-detectors and 18 light emitters, resulting in a total of 46 channels. Two 3 × 3 optode probe sets (each containing four photo-detectors and five light emitters) were used to cover the frontal and frontocentral regions as well as the parietal and occipital regions. Additionally a 3 × 5 optode probe set (containing seven photo-detectors and eight light emitters) was used to cover the central, temporal, and partially the parietal regions (Figure 2C). The probe sets were interconnected and mounted on a custom-made cap (Figure 2D). The cap was arranged in such a way that channel 40, which was used as the reference marker, was placed exactly over Cz position, according to the International 10–20 system for EEG recordings. The distance between source and detector was 3 cm, which resulted in measuring approximately 3 cm beneath the scalp. To allow a probabilistic reference to the underlying cortical areas we calculated the projections of the fNIRS channels on the cortical surface. Therefore, we used a procedure which projects topographical data based on skull landmarks into a 3D reference frame (MNI-space, Montreal Neurological Institute) optimized for fNIRS analysis (Singh et al., 2005). So for each fNIRS channel position, a set of MNI coordinates (*x*, *y*, and *z*) with an error estimated (SD) was calculated (see Figure 2E and Table 2). Table 2 shows five different regions of interest (ROI) with the according channel numbers, MNI-space correspondence (x, y, z with SD) and brodmann areas (BA). For further details on the corresponding anatomical structures see (Okamoto et al., 2004; Singh et al., 2005).

**Table 2:** **Definition and coordinates of ROIs**.

**ROI**	**Channel**	**MNI space correspondence**				**Cortical areas**	
		***x***	***y***	***z***	**SD**	**BA**	
FPI	2	−29	67	11	5	10	MFG
	4	−13	65	28	5	10	SFG
	5	−15	73	1	4	10	MeFG
FP2	9	16	67	27	5	10	SFG
	10	15	73	0	4	10	MeFG
	12	28	69	11	5	10	SFG
C3	34	−34	−7	68	8	6	PreG
	38	−47	−24	65	5	3	PosG
	43	−36	−32	72	6	4	PreG
C4	37	39	−8	68	7	6	PreG
	42	50	−24	65	5	1	PosG
	46	39	−32	71	6	4	PreG
CZ	35	−12	−4	76	7	6	SFG
	36	15	−3	76	6	6	SFG
	40	4	−18	76	8	6	MeFG
	44	−12	−32	80	6	4	PreG
	45	15	−35	80	5	4	PreG

The projections of the fNIRS channels on the cortical surface were calculated by projecting topographical data based on skull landmarks into a 3D reference frame (MNI space, Montreal Neurological Institute). The table shows five different regions of interest (ROI) with the according channel numbers, MNI space correspondence (x, y, z with SD) and brodmann areas (BA).

BA, Brodmann area; MeFG, medial frontal gyrus; MFG, middle frontal gyrus; PreG, precentral gyrus; SFG, superior frontal gyrus; PosG, postcentral gyrus.

After a visual inspection of the raw fNIRS data by a trained expert, trials containing motion artifacts were removed manually. Additionally, channels with poor signal quality, e.g. containing noise (on average less than 7% of the channels), were excluded. Baseline drifts were reduced by using a 0.01 Hz Butterworth high pass filter of order 6 with 30 dB attenuation in the stop band. Afterwards a common average reference (CAR) spatial filter was used to remove global influences like respiratory or blood pressure rhythms. As a result, for every time point, the mean of all non-excluded channels was calculated and subtracted from each channel (Pfurtscheller et al., 2010).

### Calculation of task related changes and topographic distribution

The mean task related changes of [oxy-Hb] and [deoxy-Hb] referred to a 5-s baseline interval prior the task (seconds −5 to 0) were calculated. For the excluded channels (at the maximum 6 out of 46 channels) the changes were recalculated by interpolation of the surrounding channels. In all subjects not more than one channel was interpolated in each ROI. Furthermore no interpolation was performed in frontal ROIs (FP1 and FP2). As the fNIRS data was checked for artifacts, such interpolation of channels will only cause a spatiotemporal smoothing of the hemodynamic pattern. The topographic distributions during the tasks are further visualized by plotting the [oxy-Hb] and [deoxy-Hb] values at their corresponding spatial position. A 2-D interpolation on a fine Cartesian grid was used to generate a scalp distribution. The average over two different time windows are calculated. The first time window between 0 and 4 s corresponds to the cue presentation and start of the task. The second time window between 10 and 12 s corresponds to the end of the task. The mean concentration changes of oxy-Hb and deoxy-Hb are visualized in different plots with the same scale. Increases are plotted in blue and decreases in red (according to the toolbox “EEG-Lab” from Matlab). Only well trained sequences run into analyses, concretely the mean task related concentration changes of 48 trials for each condition are plotted. The new sequences during the experimental trials were only used to keep attention.

### Statistical analyses

Before running statistical analyses the following pre-processing steps were performed:

First, five regions of interest (ROIs: FP1, FP2, C3, Cz, C4) covering the frontal and motor cortex of both hemispheres were defined: Frontal cortex: FP1 (CH: 2, 4, 5); FP2 (CH: 9, 10, 12); Motor cortex: C3 (CH: 34, 38, 43); Cz (CH: 35, 36, 40, 44, 45); C4 (CH.: 37, 42, 46). The MNI coordinates and anatomical locations of the included channels are given in Table 2 and Figure 2E. Second, the mean concentration changes were calculated in a time window of 4 s, 2 s prior and 2 s after the end of the task. Again, only the well trained sequences (48 each condition) were considered since the novel sequences were used for attentional purposes only. For statistical analyses a 2 × 2 × 2 univariate repeated measures analyses of variance (ANOVA) with the within-subject factors EXEC/INHIB (execution vs. motor inhibition), FRONTAL/CENTRAL (ROI FP1/FP2 vs. ROI C3/Cz/C4), and HEMI (left vs. right hemisphere) were applied, separately for the dependent variable oxy-Hb and deoxy-Hb and for the finger and foot movement condition.

## Results

**Figure 3:**
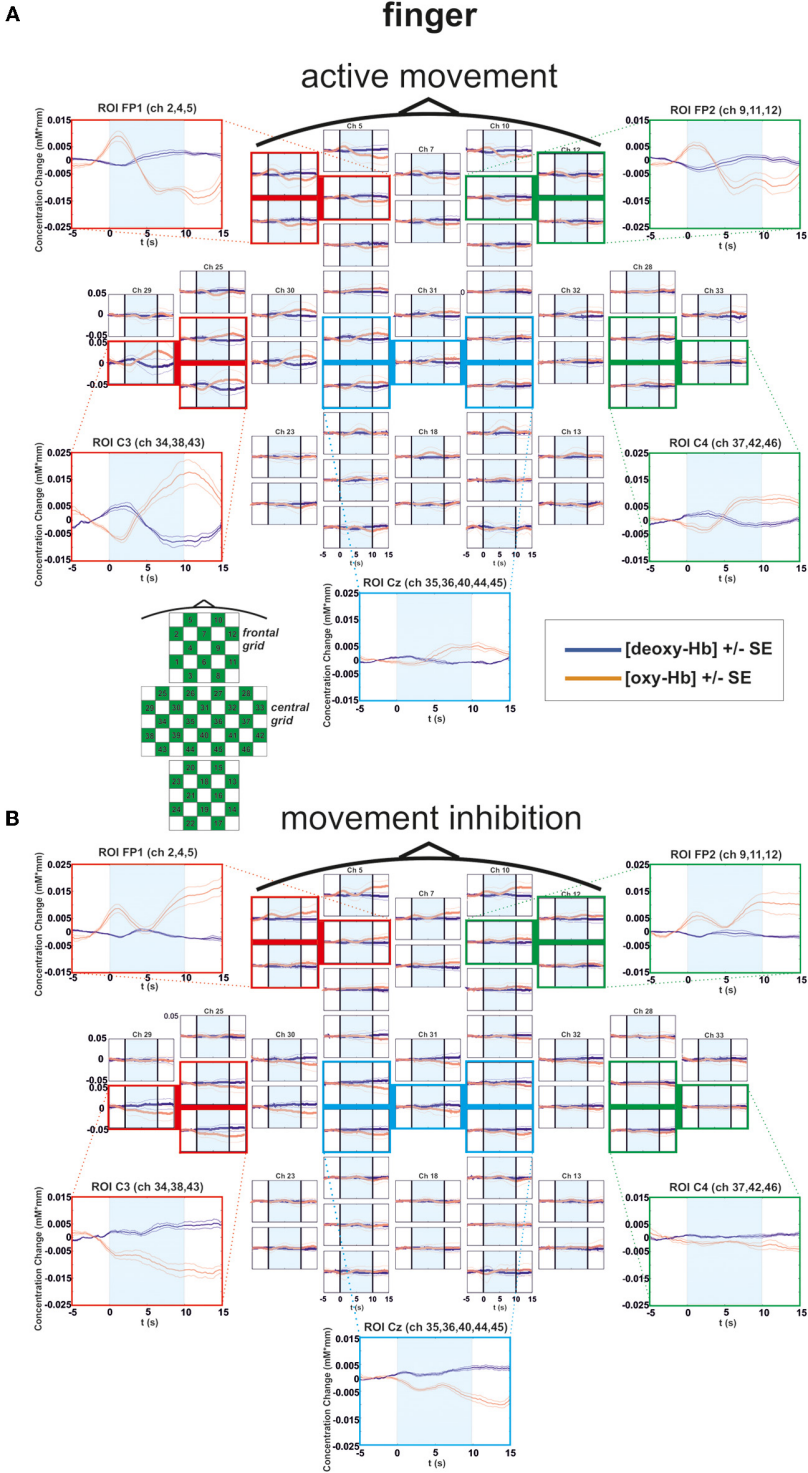
**Multichannel map illustrating oxygenation levels of ROIs of finger movement execution (A) and inhibition (B).** In the middle the mean concentration changes of [oxy-Hb] and [deoxy-Hb] for each channel are illustrated. The shaded bars indicate the activation time of 10 s. Around the channel map the defined ROIs are zoomed.

**Figure 4:**
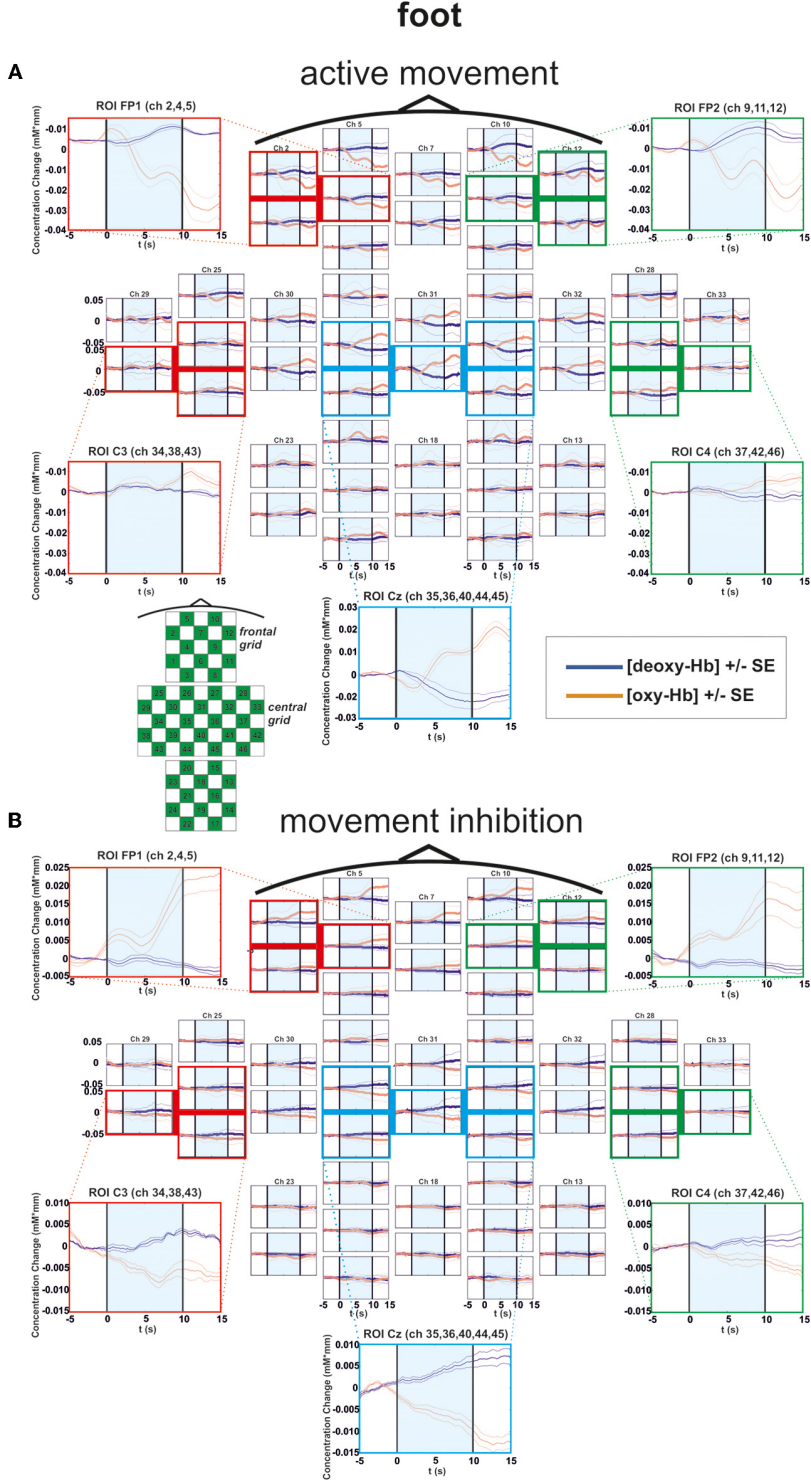
**Multichannel map illustrating oxygenation levels of ROIs of foot movement execution (A) and inhibition (B).** In the middle the mean concentration changes of [oxy-Hb] and [deoxy-Hb] for each channel are illustrated. The shaded bars indicate the activation time of 10 s. Around the channel map the defined ROIs are zoomed.

**Figure 5:**
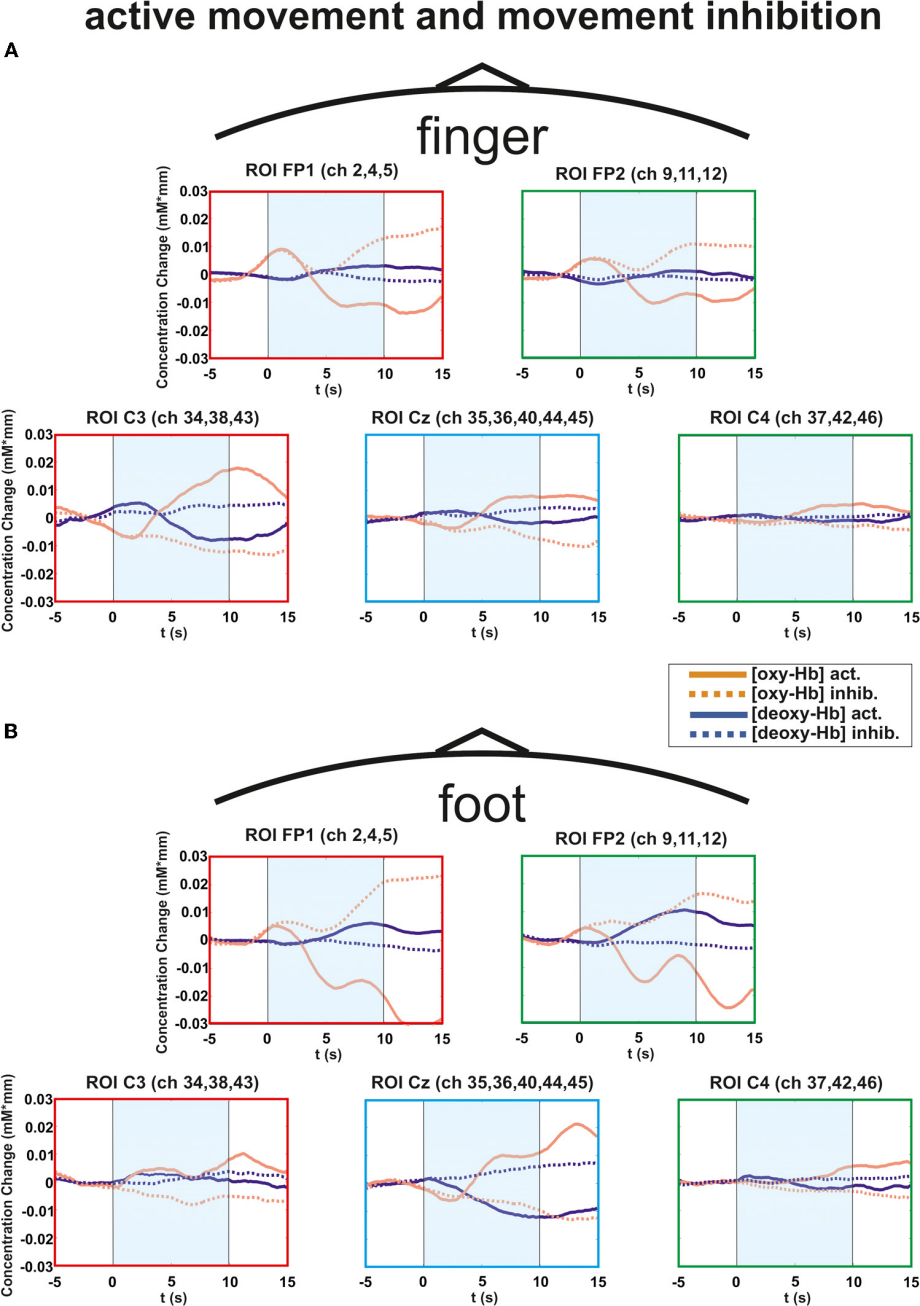
**Multichannel ROI map illustrating the mean concentration changes of [oxy-Hb] and [deoxy-Hb] for execution (thick lines) and inhibition (thin lines) together. (A)** execution/inhibition of finger movement. **(B)** execution/inhibition of foot movement. The shaded bars indicate the activation time of 10 s.

In general all subjects showed strong changes of [oxy-Hb]^2^ and [deoxy-Hb] during execution/inhibition of finger/foot movements in frontal and central cortical regions [left or medial SMA, primary motor (M1) and primary somatosensory (S1) cortex; Figures 3, 4]. During finger movement execution an [oxy-Hb] increase was found central (ROI C3) compared to the inhibition condition, where frontal regions (ROI FP1 and FP2) showed [oxy-Hb] increase and [deoxy-Hb] decrease. This effect is clearly visible in the topographic maps of Figures 5A (foot) and B (finger). Figure 5A shows oxy-Hb and deoxy-Hb concentration changes for foot movement execution (left side) and inhibition (right side) at two different points in time (0–4 s and 8–12 s). At time point 2 (8–12 s) a clear [oxy-Hb] decrease was found at central sites during execution of finger and foot movements, whereas during movement inhibition both conditions showed a [oxy-Hb] decrease at central sites and an increase at frontal sites.

In the following paragraphs significant results of the 2 × 2 × 2 univariate ANOVA are reported for finger and foot condition separately. Table 3 shows a summary of significant *F*-values for [oxy-Hb] and [deoxy-Hb]. *F*-values at 5% level are marked with one asterisk (^*^), at 1% level with two asterisks (^**^). All repeated measures tests are Huynh–Feldt corrected.

**Table 3:** **Summary of significant *F*-values for [oxy-Hb] and [deoxy-Hb]**.

**ANOVA effects (*N* = 11**)	**EXEC/INHIB (2)** × **FRONTAL/CENTRAL (2)** × HEMI (2) [oxy-Hb]	**EXEC/INHIB (2)** × **FRONTAL/CENTRAL (2)** × HEMI (2) [deoxy-Hb]
**FINGER CONDITION**		
EXEC/INHIB × FRONTAL/CENTRAL	*F*_(1, 10)_= 19.20^**^	
EXEC/INHIB × FRONTAL/CENTRAL × HEMI	*F*_(1, 10)_= 6.24^*^	*F*_(1, 10)_= 6.16^*^
**FOOT CONDITION**		
EXEC/INHIB	*F*_(1, 10)_= 10.79^**^	
EXEC/INHIB × FRONTAL/CENTRAL	*F*_(1, 10)_= 29.82^**^	*F*_(1, 10)_= 6.52^*^
FRONTAL/CENTRAL × HEMI		*F*_(1, 10)_= 5.73^*^
EXEC/INHIB × FRONTAL/CENTRAL × HEMI	*F*_(1, 10)_= 7.42^*^	

F-values at 5% level are marked with one asterisk (

* ), at 1% level with two asterisks (

** ). All repeated measures tests are Huynh–Feldt corrected.

### Finger condition

For [oxy-Hb], the ANOVA revealed a significant two-way interaction effect of EXEC/INHIB ^*^ FRONTAL/CENTRAL [*F*_(1, 10)_ = 19.20, *p* < 0.01; η^2^ = 0.66]. This interaction indicated that the type of task leads to different hemodynamic responses at frontal and central brain regions. *Post hoc*-tests (Bonferroni) showed a stronger increase in [oxy-Hb] during motor inhibition compared to active movement at frontal brain regions (FP1, FP2). At central sites (C3, Cz, C4) no significant difference in [oxy-Hb] between motor inhibition and active movement was found. Additionally [oxy-Hb] increased over central compared to frontal sites in the active movement condition. Furthermore the three-way interaction effect EXEC/INHIB ^*^ FRONTAL/CENTRAL ^*^ HEMI [*F*_(1, 10)_ = 6.24, *p* < 0.05; η^2^ = 0.38] was significant. *Post hoc-tests* (Bonferroni) showed stronger increases in [oxy-Hb] during motor inhibition compared to active movement at frontal left brain regions (FP1) and no significant difference at frontal right areas (FP2). At central left areas (C3) [oxy-Hb] was higher during active movement than during motor inhibition. Like at frontal right sites, these two conditions showed no significant difference in [oxy-Hb] at central right sites (C4). In the inhibition condition, [oxy-Hb] was significantly increased at frontal left and right compared to central left sites. In the active movement condition, [oxy-Hb] was significantly higher at central left sites than at frontal left and right sites. Summarizing, the results showed significant differences in oxy-Hb concentration changes between execution and inhibition at central and frontal sites. There is no difference in [oxy-Hb] between left and right hemisphere in the inhibition condition, leading to a more bilateral activation. The mulit-channel maps in Figure 3 showed the mean concentration changes of [oxy-Hb] and [deoxy-Hb] described above for each ROI and for execution (Figure 3A) and inhibition (Figure 3B) separately.

For [deoxy-Hb] in the finger condition, the three-way interaction effect of EXEC/INHIB ^*^ FRONTAL/CENTRAL ^*^ HEMI [*F*_(1, 10)_ = 6.16, *p* < 0.05; η^2^ = 0.38] was significant. *Post hoc*-tests (Bonferroni) indicated a stronger decrease of [deoxy-Hb] during active movement than during motor inhibition at central left brain regions (C3).

### Foot condition

In the foot condition, [oxy-Hb] was higher in the motor inhibition than in the active movement condition, which gave rise to a significant main effect of EXEC/INHIB [*F*_(1, 10)_ = 10.79, *p* < 0.01; η^2^ = 0.52]. The significant interaction effect of EXEC/INHIB ^*^ FRONTAL/CENTRAL [*F*_(1, 10)_ = 29.82, *p* < 0.01; η^2^ = 0.75] confirmed a substantial frontal increase of [oxy-Hb] during motor inhibition compared to active movement. Additionally, [oxy-Hb] was higher at frontal sites (FP1, FP2) than at central sites (Cz) in the motor inhibition condition, whereas in the active movement condition [oxy-Hb] was higher at central sites (Cz) compared to frontal areas (FP1, FP2). This effect is clearly visible in the following multi-channel map of foot movement execution (Figure 4A) and inhibition (Figure 4B). In the middle of both figures activation changes of all 52 channels are plotted. The ROI positions are illustrated in the zoomed figures around.

Furthermore, the three-way interaction effect EXEC/INHIB ^*^ FRONTAL/CENTRAL ^*^ HEMI [*F*_(1, 10)_ = 7.42, *p* < 0.05; η^2^ = 0.43] was significant, too. At central sites (Cz) no significant differences in [oxy-Hb] between active movement and motor inhibition could be found. At frontal sites (FP1, FP2), [oxy-Hb] was higher in the inhibition condition than during active movement in both hemispheres. During inhibition [oxy-Hb] was lower at central sites (left and right) compared to frontal sites (left and right). During active movement [oxy-Hb] was higher at central sites (left and right) compared to frontal sites (left and right). No significant differences in [oxy-Hb] between frontal left and right sites during inhibition were found. Again during inhibition, activation was bilateral at frontal sites like in the finger movement condition.

For [deoxy-Hb] the ANOVA revealed a significant interaction effect of EXEC/INHIB ^*^ FRONTAL/CENTRAL [*F*_(1, 10)_ = 6.52, *p* < 0.05; η^2^ = 0.39]. Like for [oxy-Hb] the type of task evokes different hemodynamic responses at frontal and central sites. Additionally, the interaction effect of FRONTAL/CENTRAL ^*^ HEMI [*F*_(1, 10)_ = 5.73, *p* < 0.05; η^2^ = 0.36] was significant, due to a stronger decrease in [deoxy-Hb] over central right sites compared to frontal right sites.

The overall results clearly show differences in the frontal and central brain regions depending on the type of task. In Figure 5 the hemodynamic responses of execution and inhibition are plotted in one graph to compare the neuronal modulations more easily. In Figure 5A the execution/inhibition responses for finger movements and in Figure 5B for foot movements are plotted together.

**Figure 6:**
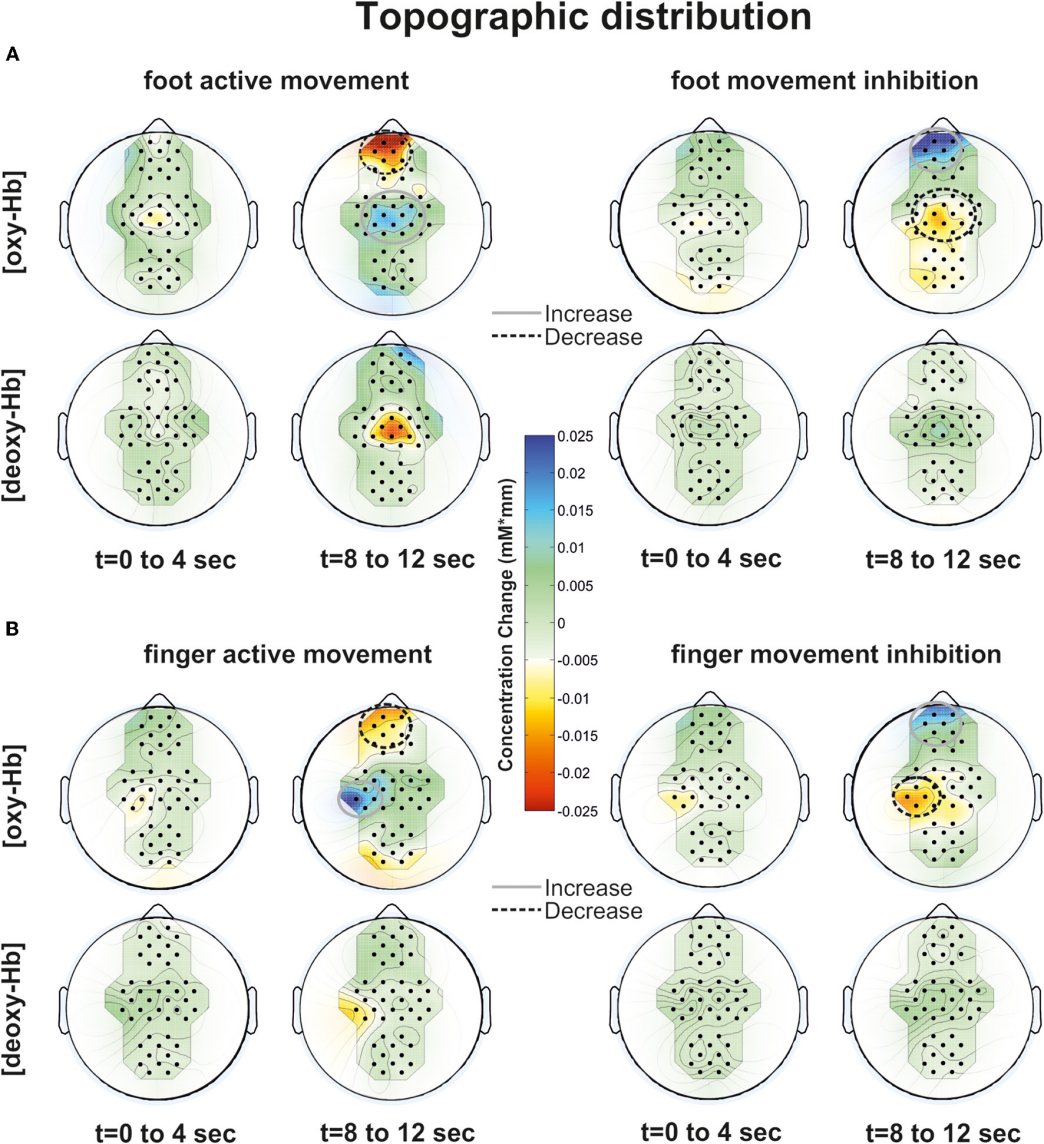
**Topographic distribution of foot (A) and finger (B) movement activation (left side) and inhibition (right side) at two time points (0–4 and 8–12 s) for [oxy-Hb] and [deoxy-Hb].** An increase of oxy/deoxy-Hb is indicated by cold colors and a decrease by warm colors.

Particularly during the finger movement execution an [oxy-Hb] increase was found centrally compared to the inhibition condition, where frontal regions showed [oxy-Hb] increase and [deoxy-Hb] decrease. This effect is clearly visible in the topographic maps of Figures 6A (foot) and B (finger). At time point 2 (8–12 s) a clear [oxy-Hb] decrease was found at central sites during execution of finger and foot movements, whereas during movement inhibition both conditions showed a [oxy-Hb] decrease at central sites and an increase at frontal sites.

## Discussion

The grand average hemodynamic response during finger movement execution showed a typical activation pattern, namely an increase in the [oxy-Hb] and a decrease of [deoxy-Hb] in the hand representation area (left sensorimotor cortex). In parallel with this activation pattern an [oxy-Hb] decrease and an increase of [deoxy-Hb] in the medial area of the anterior prefrontal cortex (APFC; approximately BA 10) was also observed. Furthermore, the responses during finger movement inhibition showed a decrease in the [oxy-Hb] and an increase of [deoxy-Hb] in the hand representation area (left sensorimotor cortex) whereas in the medial area of the APFC [oxy-Hb] increased and [deoxy-Hb] decreased. These findings are in line with previous fMRI studies (Rubia et al., 2001, 2003; Hummel et al., 2004; Nakata et al., 2008) and fNIRS studies (Boecker et al., 2007) investigating the role of the PFC during response inhibition. For example, also Rubia and colleagues (2001) found increased BOLD signals in left hemispheric dorsolateral prefrontal, medial, and parietal cortices during a go/no-go task. In a later fMRI study investigating inhibition of learned motor programs, performed by Hummel et al. (2004) was shown that the inhibitory changes are reflected by negative BOLD responses in an extended cerebro-cerebellar network of sensorimotor structures with a predominant role of the PFC.

A lot of studies identified a neural network during response inhibition consisting of ventrolateral prefrontal cortex (VLPFC), insula, basal ganglia, pre-SMA, and dorsolateral prefrontal cortex (DLPFC) (Wager et al., 2005; Aron and Poldrack, 2006; Li et al., 2008; Nakata et al., 2008; Cai and Leung, 2009; Chikazoe et al., 2009; Chen et al., 2010; Hampshire et al., 2010; Sharp et al., 2010; Tabu et al., 2011; Mirabella et al., 2012). We additionally showed that the APFC and sensorimotor regions (see also Coxon et al., 2006; Mirabella et al., 2011) are also involved. Whereas most of these studies only investigated cortical responses during execution/inhibition of hand movements, we are the first who additionally investigated metabolic changes of execution/inhibition during foot movements with fNIRS. Like the activation changes during finger movements we found the same pattern during foot movements, namely an increase of [oxy-Hb] during execution of foot movements over the corresponding representation of the sensorimotor areas regions and a further increase of [oxy-Hb] during inhibition of the same over APFC. This might be due to the interconnections of the PFC to motor areas, such as premotor, cingulate, and SMA, and to parietal areas (somatosensory areas). Another recent fMRI study performed by Tabu et al. (2012) also investigated response inhibition during hand and foot movements. They compared hand and foot inhibition mechanisms during a stop signal task. They found common inhibitory mechanisms in the pre-SMA and VLPFC regardless of modalities between hand and foot which is in line with our results.

For finger movements the same PFC activation was found in the fNIRS study by Boecker et al. (2007) using a stop-change paradigm. They compared successful as well as failed inhibition and they found PFC activation during both tasks, with pronounced activation increase in the right PFC during successful inhibition. In contrast to this study, where a two-channel fNIRS apparatus was used, we could also report activation changes over motor cortical regions additionally to PFC activity by using a multi-channel fNIRS system (46 channels). These results further support the idea that PFC activation is likely to reflect the implementation of inhibitory control of motor behavior. Covering sensorimotor areas we were able to provide evidence that appropriate contextual control of learned motor acts is represented in the brain by an extended network of sensorimotor structures in which metabolic activity is bidirectional modulated as suggested by Hummel et al. (2004). Concretely the stronger increase of [oxy-Hb] during execution compared to inhibition over sensorimotor areas and the stronger increase of [oxy-Hb] during inhibition of activation over prefrontal and SMA will support the theory of a distributed cortical network controlled by prefrontal top-down processes. The term “bidirectionality” as introduced by Hummel et al. (2004) does not stringently include causality, but rather the fact of reverse hemodynamic responses during inhibition and execution of movements. Whereas the study by Boecker et al. (2007) already showed that fNIRS is a suitable technique measuring prefrontal activation during the inhibition of initiated responses and the contribution of the PFC to response inhibition we could extend that knowledge by additionally showing a similar cortical activation pattern for execution/inhibition of foot movements with multichannel fNIRS.

The finding that inhibition/execution of learned motor programs depends on increases and decreases of neural activity in prefrontal and sensorimotor areas regardless of the effector is linked to the absence of a somatotopic organization of the PFC (see Tabu et al., 2012). Our study provides further evidence for a common neural network for finger and foot response inhibition.

All mentioned fMRI and fNIRS studies emphasize the role of the PFC during response inhibition, but in contrast to our results they primarily found activation in the right PFC. For example Boecker et al. (2007) found a substantial increase of [oxy-Hb] in the right PFC during successful inhibition of already initiated responses. For failed inhibition activation changes were observed bilaterally. Also Rubia et al. (2003) found in their event-related stop-signal study different activation patterns for successful and failed stopping. The results of the present study showed an increase of [oxy-Hb] in the APFC bilaterally for inhibition which might be due to the fact that we did not differentiate between successful and failed inhibition and both types run into analyses.

### Limitations of the study

The missing documentation of the type of inhibition is one limitation of the study which should be improved in future studies. A further limitation of the study is the lack of recording behavioral data at all. Whereas the typing frequencies of all movements have been recorded, the exact events (e.g., number of correct sequences) were missing.

## Conclusion

During finger movement execution of right handed subiects, we found an increase of [oxy-Hb] and a decrease of [deoxy-Hb] in the hand representation area (left sensorimotor cortex). Additionally a [oxy-Hb] decrease and an increase of [deoxy-Hb] in the medial area of the APFC were observed, more prominently in the left hemisphere. During finger movement inhibition a decrease in the [oxy-Hb] and an increase of [deoxy-Hb] in the hand representation area was found. Furthermore, an [oxy-Hb] increase and a [deoxy-Hb] decrease in the medial area of the APFC bilaterally and the supplementary sensorimotor regions was observed. These bidirectional neuronal control which is represented by increase/decrease of oxy-Hb and deoxy-Hb concentration are in line with the results by Hummel et al. (2004) suggesting the importance of considering not only increases but also decreases of neuronal activity in the sensorimotor network and the importance of the PFC in top-down control.

Furthermore the same interpretation is valid for foot movements, where we found an increase of [oxy-Hb] over APFC during the inhibition condition. This novel finding reinforces the claim that the PFC plays an important role during inhibitory control of motor responses (Hummel et al., 2004; Boecker et al., 2007). Clearly, inhibitory control is not a unitary process mediated by a distinct brain region, instead several neural structures contribute to different components of inhibitory control of movements. This knowledge will help to understand disorders which are closely related to inhibition, for example ADHD (Aron, 2009), bipolar disorders (Rubia et al., 2001) or Parkinson's disease (Van den Wildenberg et al., 2006; Mirabella et al., 2012).

### Conflict of interest statement

The authors declare that the research was conducted in the absence of any commercial or financial relationships that could be construed as a potential conflict of interest.

## Footnotes

^1^Subsequent the concentration of oxy-Hb and deoxy-Hb is denoted as [oxy-Hb] and [deoxy-Hb].

^2^Subsequent the concentration of oxy-Hb and deoxy-Hb is denoted as [oxy-Hb] and [deoxy-Hb].
